# Serum Prolactin Levels in Psoriasis Vulgaris

**DOI:** 10.1155/2014/586049

**Published:** 2014-02-23

**Authors:** Farhad Handjani, Nasrin Saki, Iman Ahrari, Mehdi Ebrahimi, Mohammad Mehdi Khorrami, Parastoo Nematollahi

**Affiliations:** ^1^Department of Dermatology, Molecular Dermatology Research Center, Shiraz University of Medical Sciences, Shiraz 7134844119, Iran; ^2^Department of Dermatology, Molecular Dermatology Research Center and Student Research Committee, Shahid Faghihi Hospital, Shiraz University of Medical Sciences, Zand Avenue, Shiraz 7134844119, Iran; ^3^Student Research Committee, Shiraz University of Medical Sciences, Shiraz 7134844119, Iran

## Abstract

*Background*. Psoriasis is a chronic inflammatory skin disease affecting approximately 1–3% of Caucasians. Prolactin has proliferative effects on human keratinocytes, a dominant feature of psoriasis, and it is thought that this hormone may play a role in the pathogenesis of the disease. This study was conducted to confirm or refute these findings in order to better understand the disease pathogenesis. *Methods*. The subjects were 90 individuals aged between 15 and 47 years. They were divided into three groups of 30 individuals each: psoriatic patients, atopic dermatitis patients, and control group. A questionnaire was filled regarding their demographic and medical history. All of the study subjects underwent venous blood sampling (5 mL), and serum TSH and prolactin levels were checked. Subjects with abnormal TSH were omitted. *Results*. None of the patients in the study had raised prolactin, and there was no significant difference in the serum prolactin level between patients with psoriasis and atopic dermatitis and the control group. There was no relationship between the severity of psoriasis and serum levels of prolactin. *Conclusion*. Prolactin does not seem to play a role in the pathogenesis of psoriasis as its serum levels are comparable with atopic dermatitis patients and that of the normal population.

## 1. Introduction

Psoriasis is a chronic inflammatory skin disease affecting approximately 1–3% of Caucasians [1, 2]. The diagnosis of psoriasis is mainly based on its clinical presentation. It is characterized by well-defined red, scaly plaques typically located on the scalp, knee, or elbows [3]. The main pathological features of these skin lesions are keratinocyte hyperproliferation and loss of differentiation, inflammatory cell infiltration, and vascular changes [4, 5]. Complete cure for psoriasis does not exist to date; therefore, treatment is mainly suppressive in nature and is aimed at achieving a period of remission or alleviating the severity of the disease [6].

Considering the proliferative effect of prolactin on human keratinocytes and given the fact that keratinocyte hyperproliferation is a dominant feature of psoriasis, it has been postulated that this hormone may play a role in the pathogenesis of the disease [7]. In a study from Libya, it was demonstrated that serum prolactin levels were significantly higher in patients with mild to moderate psoriasis vulgaris compared to the serum levels of prolactin in patients suffering from atopic dermatitis and those of a control group [8].

On the other hand, atopic dermatitis is also a chronic inflammatory disease associated with cutaneous hyperreactivity to environmental triggers that are innocuous to normal nonatopic individuals [9, 10]. Interestingly, genome regions of atopic dermatitis are related to psoriasis susceptibility genes, which suggest common candidate genes involved in the control of skin inflammation. Although AD and psoriasis are distinct skin diseases, both conditions involve dry, scaly skin and disrupted epidermal differentiation. Therefore, in this study we choose AD patients to compare with psoriatic patients.

As studying the etiology of psoriasis is receiving much attention nowadays and a quest for better treatment options is on the rise, this study was conducted in order to evaluate the role of prolactin in psoriasis and to compare its level between psoriatic and atopic patients.

## 2. Patients and Methods

This cross sectional case control study was performed on 90 individuals aged between 15 and 47 years which were selected randomly based on the time of referral. The individuals were divided into three groups: psoriatic patients, atopic dermatitis patients, and a control group that included healthy volunteers.

The exclusion criteria included patients who suffered from prolactinoma, thyroid disease, acromegaly, renal failure, hepatic failure, seizures, and central nervous system tumors and who were either pregnant or lactating. Patients on antipsychotics and antidepressants (including dopaminergic receptor blockers and dopamine synthesis inhibitors) were also excluded. In addition, in case any patient had used any of the following drugs: opioids, cimetidine, ranitidine, verapamil, or estrogens, at or about the time of performing the laboratory tests, he/she was excluded.

The diagnosis of psoriasis vulgaris and atopic dermatitis was confirmed by a consultant dermatologist based on clinical findings and, where indicated, by histopathological studies. Psoriasis vulgaris was the only type of psoriasis that was considered for the study and the severity of the disease was documented using PASI index. Controls were randomly selected from healthy volunteers who were matched with the study subjects in terms of gender and age. All the individuals signed the informed consent form and a questionnaire regarding their demographic data and dermatological and medical history was completed.

Venous blood sampling (5 mL) was performed and immediately centrifuged and the serum was frozen and stored until all the samples from all the cases had been collected. Using a standard kit (Prolactin IRMA 100 Test/Box—MP Biomedicals Company) and under similar laboratory conditions, serum prolactin levels for all the samples were checked and recorded by the noncompetitive immunoradiometric assay (IRMA) “sandwich type” method.

Due to the fact that persons with hypothyroidism demonstrate pseudoelevations of serum prolactin, the serum levels of TSH were also checked in all participants to rule out any subclinical or undiagnosed hypothyroidism. Abnormal values in any subject led to their omission from the study.

All data were analyzed by SPSS software 19.0 version using Kruskal-Wallis, Spearman's rho, and chi-square tests. A *P* value ≤0.05 was considered significant.

## 3. Results

The final study sample comprised of a total of 87 subjects (30 men and 56 women) as three of them were excluded due to high levels of TSH and positive history of hypothyroidism in followup and clinical investigation.

In the psoriasis group the mean prolactin level was reported as 365.05 mlU/L, with a range of 252 mlU/L to 459 mlU/L. According to the reference values of the standard kit used (66–721 for women and 30–414 for men), the laboratory's quality control reports, and consultation with the consultant endocrinologist, none of the patients in the group had increased prolactin levels. In the atopic dermatitis group, the mean serum prolactin level was 330.54 mlU/L with a range of 190–471 mlU/L, and none had an elevated serum prolactin level. In the control group, the mean serum prolactin level was 295.80 mlU/L with a range of 168.9–358.9 mlU/L. No volunteer had a high level of prolactin.

According to the Kruskal-Wallis test, there was no significant difference in the serum prolactin level among patients with psoriasis vulgaris and atopic dermatitis and the control group (*P* value = 0.604).

There was no correlation between serum prolactin level and the age of the patients in the psoriasis group. Prolactin levels appeared to decrease as the age of the patients increased in the atopic group, although this difference was not statistically significant (*P* value = 0.326). Serum prolactin, in the control group, increased with the age; however, this was not statistically significant (*P* value = 0.12).

Patients with psoriasis vulgaris had a mean TSH level of 2.32 mlU/L (1.65–2.9 mlU/L), while patients with atopic dermatitis had a mean value of 2.69 mlU/L (0.37–5.01 mlU/L), and the control group had a mean of 1.90 mlU/L (0.98–2.27 mlU/L). There were no significant differences in the serum TSH level among the three groups (*P* value = 0.080). According to the Spearman's rho test, there was no correlation between TSH and prolactin in patients with psoriasis vulgaris (correlation coefficient [*r*] = 0.050 and *P* value = 0.82).

There was no relationship between the severity of psoriasis vulgaris, as exhibited by the PASI score, and serum prolactin levels.

## 4. Discussion

The etiopathogenesis of psoriasis is still not fully understood. Recently, it has been reported that prolactin exerts a proliferative effect on human keratinocytes in vitro and may possibly play a role in the pathogenesis of psoriasis [8].

Despite the extensive list of biological activities, which includes the growth and osmoregulation of epithelial tissues as well as immunoregulatory properties, the potential significance of prolactin in human skin biology and pathology has yet to be fully appreciated [11]. Prolactin has been argued to act as a neuroendocrine modulator of skin epithelial cell proliferation and the skin immune system by forming a “prolactin-circuit” between the central nervous system and the skin [11, 12]. Binding to specific skin receptors, modulation of cytokine release in the skin, and stimulation of somatomedin release by mesenchymal cells are among the suggested pathways by which prolactin could affect epithelial cell growth in the skin. Its rise in the serum, therefore, may play a role in the hyper proliferation of keratinocytes in vivo, the hallmark of the psoriasis disease process [13]. Clinical evidence and experimental evidence in support of this theory have been postulated [13]. However, our study did not show any significant increase in prolactin levels in psoriatic or atopic patients.

Some authors have also suggested a local source of prolactin or a prolactin like substance that may induce or exacerbate psoriatic lesions which may be independent from the pituitary sources of prolactin [14]. In a study demonstrating the synthesis and release of prolactin from dermal fibroblasts in vitro, a potential local source of prolactin in the skin was suggested. It was also suggested that decidual fibroblast cells also express prolactin [15].

On the other hand, some studies have ruled out the involvement of prolactin in skin pathology and have attributed the aforementioned effects to some form of growth hormone or growth factor [12, 14]. There are studies that have proposed that the extent and severity of psoriasis correlate with growth hormone (GH) levels, although psoriatic patients, in general, have normal GH and insulin-like growth factor (IGF-1) levels [16]. One particularly interesting case report mentioned a pediatric patient with growth hormone related disease. The patient developed psoriasiform skin lesions as a possible adverse effect of GH treatment, which occurred after using higher doses (the patient also had a high IGF-1 level) [16].

With respect to AD, we did not find any evidence on the relationship between AD and serum prolactin levels. In this study, we selected AD patients along with psoriasis patients in order to compare the serum level of prolactin in each group. We included AD patients since AD and psoriasis patients have some similarities and we wanted to assess if prolactin has a special role in psoriasis as compared to AD. However, the results showed that there was no significant difference between the three groups.

## 5. Conclusion

Prolactin does not seem to play a role in the pathogenesis of psoriasis vulgaris as its serum level is comparable with atopic dermatitis patients and that of healthy controls (all within the normal range). However, studies with larger sample sizes are still necessary, to further study the role of prolactin in the pathogenesis of psoriasis.
